# Nasopharyngeal carriage of *Streptococcus pneumoniae* among HIV-infected and –uninfected children <5 years of age before introduction of pneumococcal conjugate vaccine in Mozambique

**DOI:** 10.1371/journal.pone.0191113

**Published:** 2018-02-15

**Authors:** Jennifer R. Verani, Sérgio Massora, Sozinho Acácio, Rita Teresa dos Santos, Delfino Vubil, Fabiana Pimenta, Iaci Moura, Cynthia G. Whitney, Maria Helena Costa, Eusébio Macete, Maria Benigna Matsinhe, Maria da Gloria Carvalho, Betuel Sigaúque

**Affiliations:** 1 Respiratory Diseases Branch, National Center for Immunization and Respiratory Diseases, Centers for Disease Control and Prevention, Atlanta, United States of America; 2 Centro de Investigação em Saúde de Manhiça / Fundação Manhiça, Manhiça, Moçambique; 3 Instituto Nacional de Saúde, Ministério de Saúde, Maputo, Moçambique; 4 Hospital Central de Nampula, Ministério de Saúde, Nampula Moçambique; 5 Hospital Genral de Mavalane, Ministério de Saúde, Maputo, Moçambique; 6 Ministério de Saúde, Manhiça, Moçambique; Universidade de Lisboa Faculdade de Medicina, PORTUGAL

## Abstract

Nasopharyngeal carriage is a precursor for pneumococcal disease and can be useful for evaluating pneumococcal conjugate vaccine (PCV) impact. We studied pre-PCV pneumococcal carriage among HIV-infected and -uninfected children in Mozambique. Between October 2012 and March 2013, we enrolled HIV-infected children age <5 years presenting for routine care at seven HIV clinics in 3 sites, including Maputo (urban-south), Nampula (urban-north), and Manhiça (rural-south). We also enrolled a random sample of HIV-uninfected children <5 years old from a demographic surveillance site in Manhiça. A single nasopharyngeal swab was obtained and cultured following enrichment in Todd Hewitt broth with yeast extract and rabbit serum. Pneumococcal isolates were serotyped by Quellung reaction and multiplex polymerase chain reaction. Factors associated with pneumococcal carriage were examined using logistic regression. Overall pneumococcal carriage prevalence was 80.5% (585/727), with similar prevalences among HIV-infected (81.5%, 339/416) and HIV-uninfected (79.1%, 246/311) children, and across age strata. Among HIV-infected, after adjusting for recent antibiotic use and hospitalization, there was no significant association between study site and colonization: Maputo (74.8%, 92/123), Nampula (83.7%, 82/98), Manhiça (84.6%, 165/195). Among HIV-uninfected, report of having been born to an HIV-infected mother was not associated with colonization. Among 601 pneumococcal isolates from 585 children, serotypes 19F (13.5%), 23F (13.1%), 6A (9.2%), 6B (6.2%) and 19A (5.2%) were most common. The proportion of serotypes included in the 10- and 13-valent vaccines was 44.9% and 61.7%, respectively, with no significant differences by HIV status or age group. Overall 36.9% (n = 268) of children were colonized with a PCV10 serotype and 49.7% (n = 361) with a PCV13 serotype. Pneumococcal carriage was common, with little variation by geographic region, age, or HIV status. PCV10 was introduced in April 2013; ongoing carriage studies will examine the benefits of PCV10 among HIV-infected and–uninfected children.

## Introduction

*Streptococcus pneumoniae* is a leading cause of bacterial pneumonia, meningitis and sepsis among children in developing countries[1]. An estimated 541,000 children less than 5 years of age die of pneumococcal disease each year, and most of these children live in developing countries[2]. HIV-infected children are at particularly high risk for pneumococcal disease and mortality[3, 4]. The high disease burden in developing countries, combined with limited access to health services and obstacles to providing appropriate treatment, make vaccination against pneumococcal disease a priority[5].

Pneumococcal conjugate vaccines (PCV) have been widely used in high-income countries for more than a decade, resulting in a dramatic reduction in pneumococcal disease caused by vaccine serotypes[6]. PCV has also been shown to protect against colonization with the serotypes included in the vaccine, leading to reductions in carriage and hence transmission in the community of vaccine serotypes[7–9]. Nasopharyngeal colonization with *S*. *pneumoniae* is a precursor to invasive pneumococcal infection, and the prevalence of pneumococcal carriage is very high among children, particularly in low-income countries where crowded living conditions and suboptimal sanitation facilitate transmission[10, 11]. Although most people colonized with *S*. *pneumoniae* do not develop invasive disease, examining pneumococcal carriage among young children provides insight into the serotype distribution and antimicrobial resistance of circulating strains. Nasopharyngeal carriage studies can also provide a baseline for measuring the impact of PCV introduction[12].

Mozambique introduced a 10-valent PCV (PCV10) into the national routine infant immunization schedule on April 2013. We conducted a cross-sectional nasopharyngeal carriage study to characterize *S*. *pneumoniae* carriage among HIV-infected and -uninfected children before PCV10 introduction and to determine whether carriage differed in urban and rural settings.

## Materials and methods

The study was carried out from October 2012 to March 2013 in three areas of Mozambique: Manhiça district, a rural area in the south; Maputo, an urban area in the south; and Nampula, an urban area in the north. We enrolled HIV-infected (all 3 sites) and HIV-uninfected (Manhiça only) children aged <5 years. HIV-infected children were defined as those with a positive HIV PCR at any point or positive HIV rapid test (Unigold and Determine test) after 18 months of age; they were recruited from pediatric outpatient HIV clinics in all study sites, including 4 clinics in Maputo, 3 in Nampula, and one in Manhiça. Study staff approached the parents/guardians of all children presenting to the clinics to assess eligibility. Enrollment at clinics was sequential until target enrollment numbers for each age group were achieved. HIV-uninfected children were recruited from an existing Demographic Surveillance System (DSS) in Manhiça District[13]. The DSS covers a population of approximately 92,000 people who are assigned unique identifying numbers; households within the DSS are visited twice yearly to record births, deaths, and migration. Morbidity surveillance is conducted at Manhiça District Hospital and several satellite clinics; data from healthcare facility visits and results of laboratory testing conducted at Manhiça District Hospital are linked by the DSS unique number. A stratified random sample of children aged <5 years in the DSS was selected to ensure representation across age groups (<12 months, 12–23 months and 24–59 months). Community enrollment in Manhiça was restricted to those children known to be HIV-uninfected or with unknown HIV status; because clinicians in Manhiça have a low threshold for HIV testing for children, those children with no history of HIV testing were assumed to be HIV-uninfected. For both HIV-infected and -uninfected groups, children with any sign of serious illness at the time of enrollment were excluded.

After obtaining signed informed consent from a parent or guardian, a standardized questionnaire was used to collect demographic and epidemiologic data on the study participants and their households. Nasopharyngeal specimens were collected by trained study personnel using sterile calcium alginate swabs (Fisherbrand; Fisher Scientific, Pittsburg, PA). The swabs were immediately placed in 1.0 ml skim milk-tryptone-glucose-glycerol (STGG) medium and kept on wet ice packs in a cooler box. Within 4 hours of collection, the nasopharyngeal specimens in STGG were vortexed for 10 to 20 seconds to disperse the organisms from the swab, and frozen at -70°C. For isolation, frozen specimens underwent a brief complete thawing and vigorous vortexing, then 200μl were transferred into specific broth enrichment (5.0 ml Todd Hewitt broth containing 0.5% yeast extract [THY] and 1 ml of rabbit serum), and the mixture/broth were incubated at 37C° in a CO2 incubator for 6 hours. A 10μl loop of cultured broth was inoculated on tryptone soy agar plates with 5% sheep blood (BAP), incubated at 37C° in a CO2 atmosphere and examined after 18–24 hours[14]. All suspected pneumococcal colonies were tested by optochin susceptibility (BBL Taxo; Becton Dickinson) and bile solubility 2% sodium deoxycholate (SIGMA-ALDRICH, Steinheim, Germany). When alpha-hemolytic colonies of more than one morphology were identified as possible *S*. *pneumoniae*, colonies of each morphology were selected for further testing. Pneumococcal isolates were serotyped by Quellung reaction. For non-typeable pneumococcal isolates, real time polymerase chain reaction (PCR) was performed for pneumococcal *lyt*A gene detection to confirm that the isolate was *S*. *pneumoniae*; multiplex PCR for pneumococcal serotyping was performed for *lyt*A-isolates that were non-typeable by Quellung [14, 15].

Pneumococcal isolates from HIV-infected children were tested for susceptibility to commonly used antibiotics including penicillin, ceftriaxone, amoxicillin, erythromycin, chloramphenicol, rifampicin and trimethoprim/sulfamethoxazole using the broth microdilution method. Isolates were classified as susceptible, intermediate or resistant according to the Clinical and Laboratory Standards Institute guidelines[16]; for penicillin we used both the non-meningitis parenteral breakpoints (≤2 *μ*g/mL: susceptible; 4 *μ*g/mL: intermediate; ≥8 *μ*g/mL: resistant) and the non-meningitis oral breakpoints (≤0.06 *μ*g/mL: susceptible; 0.12–1 *μ*g/mL: intermediate; ≥2 *μ*g/mL: resistant).

Statistical analysis was performed using STATA v.13 software program (StataCorp, College Station, TX, USA) and SAS v 9.3 (Cary, NC, USA). Carriage prevalence estimates and 95% confidence intervals were calculated using the binomial exact method. Association between pneumococcal carriage and other factors was examined using univariate and multivariable logistic regression. Pneumococcal vaccine serotypes were defined as those included in PCV10 (1, 4, 5, 6B, 7F, 9V, 14, 18C, 19F, and 23F) and PCV13 (PCV10 serotypes plus 3, 6A, and 19A); non-vaccine serotypes were defined as those not included in PCV10 and PCV13. Isolates with no serotype determined by Quelling and PCR were considered non-typeable.

The protocol was approved by the institutional review boards of the Mozambican Ministry of Health and the Centers for Disease Control and Prevention. Informed written consent was obtained from the parents or guardians of all participants.

## Results

**Fig 1:**
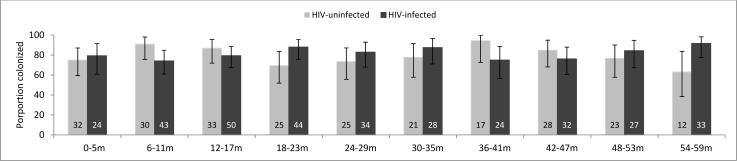
Prevalence of pneumococcal colonization among HIV-infected (n = 416) and uninfected (n = 311) children aged <5 years in Mozambique, by age group. Number at the base of the bar represents the number of children colonized within each strata.

A total of 727 children were enrolled during the study period, including 416 (57.2%) from HIV clinics and 311 (42.8%) from the DSS area (Table 1). The prevalence of nasopharyngeal carriage of *S*. *pneumoniae* was 80.5% (95% confidence interval [CI] 77.4–83.3%) overall, 81.5% (95% CI 77.4–85.1) among HIV-infected children, and 79.1% (95% CI 74.2–83.5) among HIV-uninfected children. Among HIV-uninfected children, pneumococcal carriage was similar among those whose mother reported being HIV infected during pregnancy (82.7%, 95% CI 69.7–91.8) and those whose mother was reportedly HIV-negative (79.8%, 95% CI 73.3–85.3). Carriage prevalence was also consistent between 249 children with a documented negative HIV test (78.7%, 95%CI 73.1–83.6) and 62 children assumed to be HIV-uninfected (80.7%, 95%CI 68.6–89.6)The similarity in colonization prevalence among HIV-infected and–uninfected children was consistent across age groups (Fig 1).

**Table 1:** Prevalence of pneumococcal carriage by HIV infection, HIV exposure, and site.

Variable	Number tested	Number with *S pneumoniae* detected	Prevalence	95% Confidence interval
HIV status				
Uninfected	311	246	79.1	74.2 to 83.5
Infected	416	339	81.5	77.4 to 85.1
HIV exposure, HIV-uninfected*				
Exposed	52	43	82.7	69.7 to 91.8
Unexposed	188	150	79.8	73.3 to 85.3
Site, HIV-infected				
Manhica	195	165	84.6	78.8 to 89.4
Maputo	123	92	74.8	66.2 to 82.2
Nampula	98	82	83.7	74.8 to 90.4
**Overall**	**727**	**585**	**80.5**	**77.4 to 83.3**

* HIV exposure data reported by parent/guardian; missing for 72/311 (23%)

Among HIV-infected children, the prevalence of pneumococcal carriage was lower in Maputo (74.8%) than in the other two sites (Table 1, 84.6% in Manhiça and 83.7% in Nampula); however on multivariable analyses (Table 2), there was no significant association between site and pneumococcal colonization. Hospitalization within the past 3 months (adjusted odds ratio [aOR] 0.364, 95% CI 0.195, 0.681), and reported antibiotic use within the past 3 weeks (aOR 0.472, 95% CI 0.224, 0.997) were significantly less common among colonized children compared with those not colonized with *S*. *pneumoniae*. No risk factors for pneumococcal colonization were identified.

**Table 2:** Factors associated with pneumococcal colonization among HIV-infected and–uninfected children aged <5 years in Mozambique.

Characteristic	HIV-infected				HIV-uninfected		
	Colonized (n = 339) n (%)	Not colonized (n = 77) n (%)	Crude odds ratio (95% confidence interval)	Adjusted odds ratio (95% confidence interval)	Colonized (n = 246) n (%)	Not colonized (n = 65) n (%)	Crude odds ratio (95% confidence interval)
Male sex	168 (49.6)	31 (40.3)	1.458 (0.882, 2.410)		118 (48.0)	30 (46.2)	1.076 (0.622, 1.861)
Age <24 months	161 (47.5)	41 (53.2)	0.794 (0.484, 1.304)		120 (48.8)	30 (46.2)	1.111 (0.642, 1.922)
Malnutrition							
None	171 (50.7)	34 (45.3)			170 (70.3)	43 (69.4)	
Mild	86 (25.5)	13 (17.3)	1.315 (0.660, 2.621)		47 (19.4)	15 (24.2)	0.793 (0.405, 1.550)
Moderate	45 (13.4)	17 (22.7)	0.526 (0.270, 1.027)		18 (7.4)	4 (6.4)	1.138 (0.366, 3.537)
Severe	35 (10.4)	11 (14.7)	0.633 (0.293, 1.368)		7 (2.9)	0 (0)	
Ever breastfed	307 (93.9)	74 (97.4)	0.415 (0.095, 1.814)		233 (97.9)	60 (96.8)	1.554 (0.294, 8.207)
Currently breastfeeding	78 (24.5)	19 (27.1)	0.869 (0.484, 1.560)		87 (37.3)	19 (30.2)	1.380 (0.757, 2.514)
Current symptoms							
Fever	17 (5.0)	3 (4.0)	1.289 (0.368, 4.513)		17 (6.9)	2 (65.0)	2.349 (0.529, 10.436)
Cough	44 (13.0)	6 (7.9)	1.746 (0.716, 4.260)		41 (16.7)	12 (18.5)	0.888 (0.436, 1.807)
Runny nose	147 (43.8)	31 (41.9)	1.079 (0.648, 1.796)		103 (42.2)	21 (32.8)	1.506 (0.843, 2.692)
Hospitalization in past 3 months	46 (13.7)	29 (38.7)	0.252 (0.144, 0.442)	**0.364 (0.195, 0.681)**	3 (1.3)	0 (0)	
Antibiotic use in past 3 weeks	28 (8.4)	18 (24.3)	0.286 (0.148, 0.551)	**0.472 (0.224, 0.997)**	18 (7.4)	0 (0)	
Attends school	10 (3.0)	4 (5.3)	0.555 (0.169, 1.819)		5 (2.1)	1 (1.6)	1.369 (0.157, 11.931)
Smokers in house	38 (12.1)	9 (12.2)	0.994 (0.458, 2.159)		35 (15.0)	11 (17.2)	0.852 (0.405, 1.789)
≥6 additional persons living in house	166 (49.6)	38 (50.0)	0.982 (0.597, 1.616)		149 (60.6)	36 (55.4)	1.237 (0.713, 2.149)
Cook with charcoal/wood	292 (91.3)	72 (97.3)	0.290 (0.067, 1.244)		211 (93.4)	60 (93.75)	0.938 (0.300, 2.931)
Walls made of mud or sticks	143 (43.3)	34 (44.7)	0.945 (0.572, 1.560)		123 (51.3)	27 (42.86)	1.402 (0.801, 2.453)
HIV exposure	—	—	—		43 (22.3)	9 (19.15)	1.210 (0.543, 2.699)
Antiretroviral therapy	258 (76.1)	57 (74.0)	1.118 (0.634, 1.971)		—	—	—
Cotrimoxazole prophylaxis	264 (79.0)	66 (86.8)	0.571 (0.279, 1.169)		—	—	—
Site							
Manhiça	165 (48.7)	30 (39.0)	reference		—	—	—
Maputo	92 (27.1)	31 (40.3)	0.540 (0.307, 0.948)		—	—	—
Nampula	82 (24.2)	16 (20.8)	0.932 (0.481, 1.807)		—	—	—

**Fig 2:**
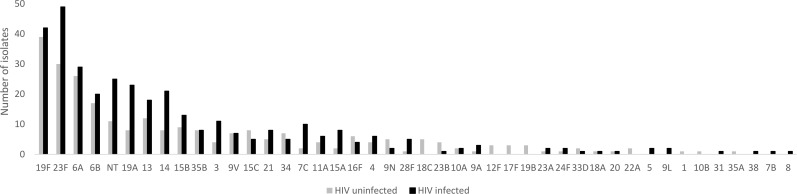
Serotype distribution among nasopharyngeal *Streptococcus pneumoniae* isolates (n = 601) found among HIV-infected and -uninfected children aged <5 years in Mozambique.

A total of 601 pneumococcal isolates were obtained for serotyping, including isolates of 1 serotype from 569 children plus isolates of 2 different serotypes from each of 16 children. Among 43 different serotypes identified, the most frequent were 19F (n = 81, 13.5%), 23F (n = 79, 13.1%), 6A (n = 55, 9.2%), 6B (n = 37, 6.2%) and 19A (n = 31, 5.2%); 36 (6.0%) isolates were non-typeable. Although some differences were noted in the distribution of serotype by HIV status (Fig 2), only the prevalence of 18C was found to be significantly different (0% among HIV-infected vs. 2.0% among HIV-uninfected; p = 0.014).

Among 601 isolates, 44.9% (n = 270) were serotypes included in PCV10, and 61.7% (n = 371) were serotypes included in PCV13. Among all 727 children, 36.9% (n = 268) were colonized with a PCV10 serotype and 49.7% (n = 361) with a PCV13 serotype. The proportion of children colonized with a vaccine serotype did not significantly vary by HIV status for either PCV10 (37.5% in HIV-infected, 36.0% among HIV-uninfected; p = 0.681) or for PCV13 (51.7% and 47.0%, respectively; p = 0.206). The proportion colonized with a vaccine serotype also did not vary significantly by age for either PCV10 (34.9% in <2 year olds, 38.7 in ≥2 year olds; p = 0.298) or PCV13 (48.6% in <2 year olds, 50.7% in ≥2 year olds; p = 0.574). The proportion colonized with a vaccine serotype was somewhat higher in the northern region (Nampula), compared to other sites; for PCV13 the difference was significant (62.2% in Nampula, 47.7% in other sites; p = 0.007).

A total of 346 isolates were obtained from 339 HIV-infected children with pneumococcal colonization; 343 (99.1%) underwent antimicrobial susceptibility testing (Table 3). Resistance to penicillin was <1%. The frequency of intermediate susceptibility to penicillin varied depending upon the breakpoints used; this proportion was <1% using parenteral (non-meningitis) breakpoints and 65.3% using oral breakpoints. No isolates were resistant to ceftriaxone; one was resistant to amoxicillin and two were resistant to rifampin. Resistance to trimethoprim-sulfamethoxazole was common (88.6%). Among 307 isolates resistant to at least one of the antibiotics tested, 47.6% (n = 146) were PCV10 serotypes and 66.8% (n = 205) were PCV13 serotypes.

**Table 3:** Antibiotic susceptibility among pneumococcal isolates (n = 343) obtained from HIV-infected children.

Antimicrobial agents	Susceptible n (%)	Intermediate n (%)	Resistant n (%)
Penicillin, non-meningitis parenteral	340 (99.1)	3 (0.9)	0 (0)
Penicillin, non-meningitis oral	116 (33.8)	224 (65.3)	3 (0.9)
Trimethoprim-Sulfamethoxazole	15 (4.4)	24 (7.0)	304 (88.6)
Ceftriaxone	343 (100.0)	0 (0.0)	0 (0.0)
Amoxicillin	339 (98.8)	3 (0.9)	1 (0.3)
Erythromycin	286 (83.4)	1(0.3)	56 (16.3)
Chloramphenicol	320 (93.3)	0 (0.0)	23 (6.7)
Rifampin	341 (99.4)	0 (0.0)	2 (0.6)

MIC interpretative Criteria according with Clinical and Laboratory Standard Institute (2013).

## Discussion

We observed high rates of pneumococcal colonization among children aged less than five years prior to PCV10 introduction in Mozambique, with approximately four out of five children carrying at least one pneumococcal serotype. There was remarkably little variation in the prevalence of pneumococcal carriage; we found no significant differences between HIV-infected and -uninfected, between three distinct geographic and epidemiologic contexts, and across age strata among children less than five years. The most common colonizing serotypes are either included in PCV10, which was introduced in 2013, or are serotypes against which PCV10 may provide cross-protection. The serotype coverage of PCV13 (61.7%) was higher than that of PCV10 (44.9%), primarily because of the inclusion of serotypes 6A and 19A in PCV13. These data provide useful insight into the potential impact of PCV10 introduction in Mozambique.

The colonization prevalence we observed was higher than that seen in many other African settings. A recent review of published literature on pneumococcal carriage in sub-Saharan Africa found an overall prevalence of 63% among children less than five years, with individual studies reporting a range of 21% to 94%[10]. The broth enrichment used in this study, Todd Hewitt with the addition of yeast extract and rabbit serum, has been shown to increase the detection of *S*. *pneumoniae* in nasopharyngeal specimens[14]. Concerns have been raised about potential disadvantages of using broth enrichment, including overgrowth of other species and differential rates of growth among different pneumococcal serotypes[17]. However a recently published study of pneumococcal carriage among children under five years in Kenya that used the same broth enrichment reported a prevalence of 90% (reflecting 57 pneumococcal serotypes)[15], while other carriage studies from Kenya have found 22% to 74% of children less than five years to be colonized[18–21]. A previous study of pneumococcal carriage in Mozambique that did not employ broth enrichment isolated pneumococci from 87% of enrolled children[22]; however that study was conducted among children attending an outpatient clinic and thus included some with acute respiratory infection, which is a known risk factor for pneumococcal colonization[23]. In our analysis of risk factors, we did not identify any variables significantly associated with colonization among HIV-uninfected children; however the power of the analysis was limited because so few children were not colonized. Among HIV-infected children, we found that recent antibiotic use and hospitalization within the past 3 months, which may be a proxy for antibiotic use, were protective against colonization. The prevalence of colonization was lower in Maputo compared with the other two sites; however after adjusting for recent hospitalization and antibiotic use, there was no significant association between colonization and study site.

We found no significant difference in the prevalence of colonization by HIV status, in contrast to a study in Kenya that reported a significant association between HIV infection and pneumococcal carriage among children[18]. Furthermore, among HIV-uninfected children, the proportion colonized was similar among those exposed to HIV in-utero and those born to HIV-uninfected mothers. Although there were some differences observed in the serotype distribution among isolates from HIV-infected and–uninfected children, there was no significant difference in the serotype coverage of available PCVs. HIV is an important risk factor for pneumococcal disease, with rates of invasive pneumococcal disease among HIV-infected children up to 43 times those of HIV-uninfected children[3, 24]. Data on PCV effectiveness against pneumococcal disease among HIV-infected children are limited[25–27]. If the vaccine is less protective in HIV-infected children, they might continue to be at high risk for pneumococcal disease, and potentially serve as a reservoir for vaccine serotypes, even after PCV introduction. It is important to monitor PCV impact on carriage and disease in settings with a high HIV burden as well as to continue to support measures to prevent HIV infections.

Our data suggest that PCV will have an important impact in Mozambique. The two most common colonizing serotypes, 19F and 23F, comprise more than a quarter of all colonizing isolates and are included in both PCV10 and PCV13. Other leading serotypes, such as 6A and 19A, are included in PCV13 but not in PCV10. Immunogenicity data from pre-licensure clinical trials suggested that PCV may provide cross-protection against these serotypes[28, 29]. However, data on PCV10 impact on 6A and 19A carriage and disease are inconclusive. A case-control study of PCV10 effectiveness against invasive pneumococcal disease in Brazil found significant protection against serotype 19A[30], and an analysis of incidence trends in Finland following PCV10 introduction identified significant declines in invasive disease caused by serotypes 6A and 19A[31]. However, studies from Kenya[21] and Brazil[32] showed no significant change in the prevalence of carriage of serotypes 6A and 19A after PCV10 implementation. A randomized controlled trial in Finland found a significant reduction in carriage of 19A among children receiving 4 doses of PCV10 (3 primary doses and a booster), but not among children receiving 3 doses (2 primary doses and a booster)[33]; this same study reported no impact of PCV10 on carriage of serotype 6A. An ongoing study examining the impact of PCV10 introduction on carriage in Mozambique will provide addition insight into potential cross-protection of PCV10 against serotypes 6A and 19A.

Penicillin-resistance among *S*. *pneumoniae* has been an increasing global concern since it was first reported in the late 1960s[34]. In this study, colonizing pneumococcal isolates were rarely resistant to penicillin or amoxicillin, while the frequency of intermediate susceptibility to penicillin varied greatly (<1% or 65.3%) depending upon the breakpoints used. Our results are similar to those of a recent study in Kenya that used oral breakpoints and found that 79% of colonizing pneumococcal isolates demonstrated intermediate susceptibility to penicillin and 2.4% were resistant[15]. A review of antimicrobial resistance among *S*. *pneumoniae* isolates in Africa reported 25% non-susceptibility to penicillin (using meningitis breakpoints) and 26% non-susceptibility to ampicillin among colonizing isolates[35]. Our finding of infrequent resistance to beta lactams in Mozambique supports the current first line treatment recommendations for use of beta lactams to treat child pneumonia. However, given the prevalence of intermediate penicillin susceptibility using oral breakpoints, continued monitoring for increased penicillin resistance is warranted. The prevalence of trimethoprim-sulfamethoxazole non-susceptibility in this study (>95%) was higher than that reported by the above-mentioned review (47.5%), and similar to the recent study from Kenya (98%). The high level of trimethoprim-sulfamethoxazole resistance in Mozambique and Kenya likely reflects frequent use of trimethoprim-sulfamethoxazole for prophylaxis in settings with a high burden of HIV.

This study has several limitations. The HIV-infected participants were selected as a convenience sample of those who attended the HIV clinics for either routine care or management of an acute illness during the study enrollment period; they may differ from HIV-infected children who did not seek care at the clinics. HIV-uninfected children were enrolled from only one site, Manhiça, limiting our ability to generalize results to other areas of Mozambique. However, among the HIV-infected children, we observed very little variation in pneumococcal colonization across different geographic regions. Although most of the children in the HIV-uninfected group had a documented negative HIV test, some were assumed to be uninfected because they never underwent HIV testing and the threshold for testing infants for HIV in the study setting is low; it is possible that some children presumed to HIV-uninfected were actually infected. In addition, for HIV-uninfected children, data on HIV exposure in-utero was reported by the parent/guardian, which could have resulted in misclassification. Because of limited resources, antimicrobial susceptibility testing was only performed for colonizing isolates from HIV-infected children. Susceptibility patterns may differ among HIV-uninfected children. Certain PCV serotypes known to be important causes of invasive disease in Mozambique, such as serotypes 1 and 5, are rarely detected in carriage[22]. Thus, descriptions of the serotype coverage of PCV formulations are an underestimate of the potential impact the vaccine could have on pneumococcal disease.

This cross-sectional study demonstrated a high prevalence of pneumococcal carriage among children under the age of five years in Mozambique. Colonization did not vary by age, geographic region, HIV-infection or HIV exposure. These data can serve as a baseline for evaluating PCV10, which was introduced in April 2013. Vaccine impact on carriage in Mozambique will depend on potential cross-protection against serotypes 6A and 19A, and the impact against disease will depend on vaccine effectiveness against serotypes not reflected in carriage data (serotypes 1 and 5). Our ongoing work to evaluate PCV10 impact on carriage, pneumonia and invasive disease will help address questions about PCV performance in high-HIV prevalence settings with a high burden of pneumococcal disease.

## Supporting information

S1 Table Anonymized dataset Mozambique pneumococcal carriage pre-PCV. (XLS)
